# Age-Related Vascular Changes Affect Turbulence in Aortic Blood Flow

**DOI:** 10.3389/fphys.2018.00036

**Published:** 2018-01-25

**Authors:** Hojin Ha, Magnus Ziegler, Martin Welander, Niclas Bjarnegård, Carl-Johan Carlhäll, Marcus Lindenberger, Toste Länne, Tino Ebbers, Petter Dyverfeldt

**Affiliations:** ^1^Department of Mechanical and Biomedical Engineering, Kangwon National University, Chuncheon, South Korea; ^2^Division of Cardiovascular Medicine, Department of Medical and Health Sciences, Linköping University, Linköping, Sweden; ^3^Center for Medical Image Science and Visualization, Linköping University, Linköping, Sweden; ^4^Department of Thoracic and Vascular Surgery, Linköping University, Linköping, Sweden; ^5^Department of Clinical Physiology, Department of Medical and Health Sciences, Linköping University, Linköping, Sweden; ^6^Department of Cardiology, Linköping University, Linköping, Sweden

**Keywords:** turbulent kinetic energy (TKE), turbulent blood flow, aortic blood flow, aortic dilation, normal values, 4D flow MRI, phase contrast MRI

## Abstract

Turbulent blood flow is implicated in the pathogenesis of several aortic diseases but the extent and degree of turbulent blood flow in the normal aorta is unknown. We aimed to quantify the extent and degree of turbulece in the normal aorta and to assess whether age impacts the degree of turbulence. 22 young normal males (23.7 ± 3.0 y.o.) and 20 old normal males (70.9 ± 3.5 y.o.) were examined using four dimensional flow magnetic resonance imaging (4D Flow MRI) to quantify the turbulent kinetic energy (TKE), a measure of the intensity of turbulence, in the aorta. All healthy subjects developed turbulent flow in the aorta, with total TKE of 3–19 mJ. The overall degree of turbulence in the entire aorta was similar between the groups, although the old subjects had about 73% more total TKE in the ascending aorta compared to the young subjects (young = 3.7 ± 1.8 mJ, old = 6.4 ± 2.4 mJ, *p* < 0.001). This increase in ascending aorta TKE in old subjects was associated with age-related dilation of the ascending aorta which increases the volume available for turbulence development. Conversely, age-related dilation of the descending and abdominal aorta decreased the average flow velocity and suppressed the development of turbulence. In conclusion, turbulent blood flow develops in the aorta of normal subjects and is impacted by age-related geometric changes. Non-invasive assessment enables the determination of normal levels of turbulent flow in the aorta which is a prerequisite for understanding the role of turbulence in the pathophysiology of cardiovascular disease.

## Introduction

Turbulent blood flow in the human body is linked to the pathogenesis of various cardiovascular diseases. Turbulence increases the fluid-dynamic shear stress on blood constituents, and promotes platelet aggregation leading to thrombus development in disturbed flow regions such as those downstream from stenotic aortic valves (Mustard et al., 1962; Smith et al., 1972; Stein et al., 1977, 1982; Yoganathan et al., 1986). Similarly, elevated shear stress exceeding the hemolytic threshold can damage or destroy red blood cells (Sallam and Hwang, 1983; Lu et al., 2001; Yen et al., 2014). Turbulent blood flow also influences the endothelial cell lining of the vessel wall through mechanotransduction mechanisms (Humphrey et al., 2015), and is implicated in the initiation, progression, and development of atherosclerosis (Davies et al., 1986; Davies, 2009; Mehta and Tzima, 2016; Wang et al., 2016).

Various acquired and congenital cardiovascular disorders, such as aortic valve stenosis and aortic coarctation, contribute to elevated levels of turbulent flow in the aorta (Stein and Sabbah, 1976; Yamaguchi et al., 1988; Dyverfeldt et al., 2013; Lantz et al., 2013). Turbulence appears to negatively impact several biological tissues. However, while it is well-known that turbulence is present in patients with obstructive disease in the major vessels, the extent of turbulence in aortic blood flow in healthy normal subjects is relatively unexplored. Interestingly, catheter-based measurements of human and dog blood flow have revealed that turbulence indeed can develop in the aorta not only with diseased but also with normal aortic valves (Stein and Sabbah, 1976; Yamaguchi et al., 1983; Hanai et al., 1991). Although limited by a small sample size, these previous studies raise the question of the extent to which turbulence is present in normal aortic flow and how it might be impacted by aortic diameter, patient age, etc. Determining the normal or expected extent and degree of turbulent flow in the aorta is a prerequisite for understanding the role of turbulence in the pathophysiology and clinical risk-stratification of pathological conditions in the cardiovascular system.

Age-related changes in vascular structure and function influence the physiological flow patterns in the aorta. For example, normal aging brings an increase of collagen, a reduction of elastin content, and calcification, which results in a more stiff and dilated vessel (Lakatta and Levy, 2003). Consequently, hemodynamic parameters such as velocity and wall shear stress change with age (Brandfonbrener et al., 1955; Van Ooij et al., 2015). Therefore, we hypothesized that the physiological level of turbulence in the aortic blood flow of normal subjects changes with age. Specifically, given that the prevalence of vascular disease increases with age (Savji et al., 2013), older subjects can be expected to have more pathological aortic blood flow, as characterized by elevated turbulence, when compared to young subjects.

Accordingly, the aim of this study was to characterize the extent and degree of turbulence in young and old healthy subjects to determine whether or not turbulence is present in the normal aorta. We hypothesized that the amount of turbulence is affected by age-related changes in vascular anatomy and function. In an attempt to identify determinants of turbulence in aortic blood flow, vessel diameters and conventional flow parameters were characterized.

## Materials and methods

### Study population

The ethical review board in Linköping, Sweden approved the study and all subjects gave written informed consent. Between September 2015 and December 2016, a total of 47 subjects were recruited and divided into two study groups: 1) young normal subjects (from hereon referred to as “young”) recruited from the medical school at Linköping University, 2) old normal subjects (from hereon referred to as “old”) recruited from a surveillance program at Linköping University Hospital. Inclusion criteria for (I) all subjects: sinus rhythm, absence of contraindications for MRI; (II) young: age 18–30 years; (III) old: age 66–76 years; (IV) young and old: absence of current cardiovascular disease, diabetes and smoking. After excluding the cases with an electrocardiogram (ECG) gating error (*n* = 1), suspension due to high peripheral nerve stimulation (PNS, *n* = 1), and missing 4D Flow MRI protocol (*n* = 3), a total of 42 subjects (all male) were included in the study (young, *n* = 22; old, *n* = 20).

### Data acquisition and quantification of TKE

Recent advances in magnetic resonance (MR) flow imaging permit non-invasive quantification of turbulent blood flow *in-vivo* through measurements of the turbulent kinetic energy (TKE) (Dyverfeldt et al., 2006, 2008). While conventional MR velocity mapping techniques measure spatiotemporally averaged velocity fields, TKE describes the energy content of turbulent flows and is a measure of the intensity of turbulent velocity fluctuations.

Time-resolved, three-dimensional phase-contrast MRI data with three-directional motion-encoding (4D Flow MRI) were acquired on a clinical 3T MRI scanner (Philips Ingenia; Philips Healthcare, Best, the Netherlands) using a retrospectively cardiac-gated gradient-echo sequence with four-point asymmetric flow encoding, where the latter enables measurements of TKE (Dyverfeldt et al., 2015). The 4D Flow MRI data were acquired post injection of a Gd contrast agent (Magnevist, Bayer Schering Pharma AG). The scans were performed during free breathing and respiratory effects were suppressed using navigator gating. Scan parameters included: VENC = 100–200 cm/s, flip angle 15°, echo time = 2.5–3.1 ms, repetition time = 4.4–5.0 ms. The acquired temporal resolution was 35–40 ms. The 3D field of view (FOV) = 480–560 × 480–560 × 71–117 mm^3^ and matrix size = 192–224 × 192–224 × 28–46 were adjusted depending on each subject's anatomy to cover the whole aorta from the aortic valve to the iliac bifurcation with a sagittal-oblique slab orientation while maintaining an isotropic voxel size of 2.5 × 2.5 × 2.5 mm^3^. Total scan time was 10–15 min including respiratory navigator gating.

Mean velocity fields were reconstructed from the 4D Flow MRI data using conventional phase-difference algorithms. Corrections were made for concomitant gradient field effects, phase-wraps (Wigström et al., 1999), and background phase-offsets (Ebbers et al., 2008).

**Figure 1:**
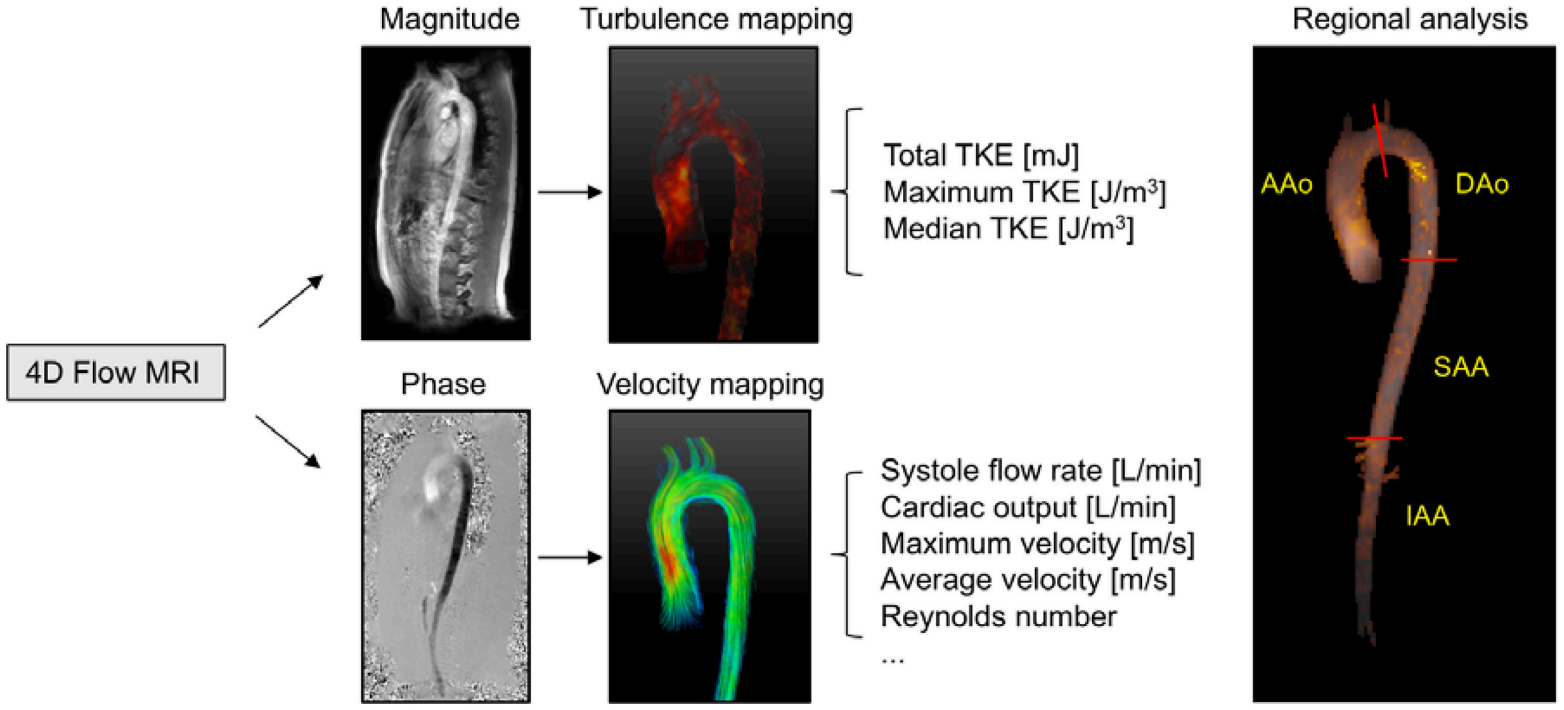
Schematic overview of hemodynamic analysis using 4D Flow MRI. 4D Flow MRI acquires the turbulence and velocity data from the magnitude and phase images, respectively. Regional analyses were performed in four different anatomical regions (AAo, ascending aorta; DAo, descending aorta; SAA, suprarenal abdominal aorta; IAA, infrarenal abdominal aorta).

The magnitude images of the individual flow-encoding segments were reconstructed to compute the TKE (Figure 1). The TKE is defined as (Mathieu and Scott, 2000):

(1) $$TKE=\;\frac{1}{2}\rho \sum_{i\;=\;1}^{3}\sigma_{i}^{2}\;[J/m^{3}]$$

where ρ is the fluid density, and σ_*i*_ is the velocity fluctuation intensity in three orthogonal directions. For asymmetric four - point flow encoding, as used here, σ_*i*_, is obtained as (Dyverfeldt et al., 2009, 2013):

(2) $$\sigma_{i}=\frac{1}{k_{v}}\sqrt{2\frac{\left|S\right|}{\left|S_{i}\right|}}$$

where |S_i_| and |S| are the magnitude of MR signal with and without motion sensitivity encoding, respectively. k_v_ (= π/*VENC*) describes the motion sensitivity.

### Data analysis

Vessel diameter, maximum flow rate, Reynolds numbers, and TKE were computed for the entire aorta as well as four regional aortic segments. The regional aortic segments were: (1) the ascending aorta (AAo), defined as the region from the aortic valve level to the middle of the aortic arch; (2) the descending aorta (DAo), defined as the region from the middle of the aortic arch to the distal descending aorta at the same level with the aortic valve; (3) the suprarenal abdominal aorta (SAA), defined as the region from the distal descending aorta to the renal artery branches; and, (4) the infrarenal abdominal aorta (IAA), defined as the region from the renal artery branching to the iliac bifurcation (Figure 1). Geometric constraints were obtained through semi-automatic segmentation of the 4D Flow MRI data.

Vessel diameter and flow rate were extracted at 1 mm intervals throughout the aorta using previously described centerline methods (Dyverfeldt et al., 2014). The segment-averaged diameter (D) and peak flow rate (Q_max_) were calculated from time-averaged angiographic data, yielding D_AAo_, D_DAo_, D_SAA_, D_IAA_, and Q_max_, _AAo_, Q_max_, _DAo_, Q_max_, _SAA_, Q_max_, _IAA_. Stroke volume (SV) was calculated by integrating the flow rate in the ascending aorta through the cardiac cycle and cardiac output (CO) was calculated as the product of SV and heart rate. The maximum Reynolds number (Re) was calculated for each aortic segment as: Re_max_ = Q_max_D/νA, where Q_max_ is the maximum flow rate, D is the diameter, ν is the kinematic viscosity, and A is the cross-sectional area. The kinematic viscosity was assumed to be 3.77 × 10^−6^ m^2^/s (Dintenfass, 1985).

**Figure 2:**
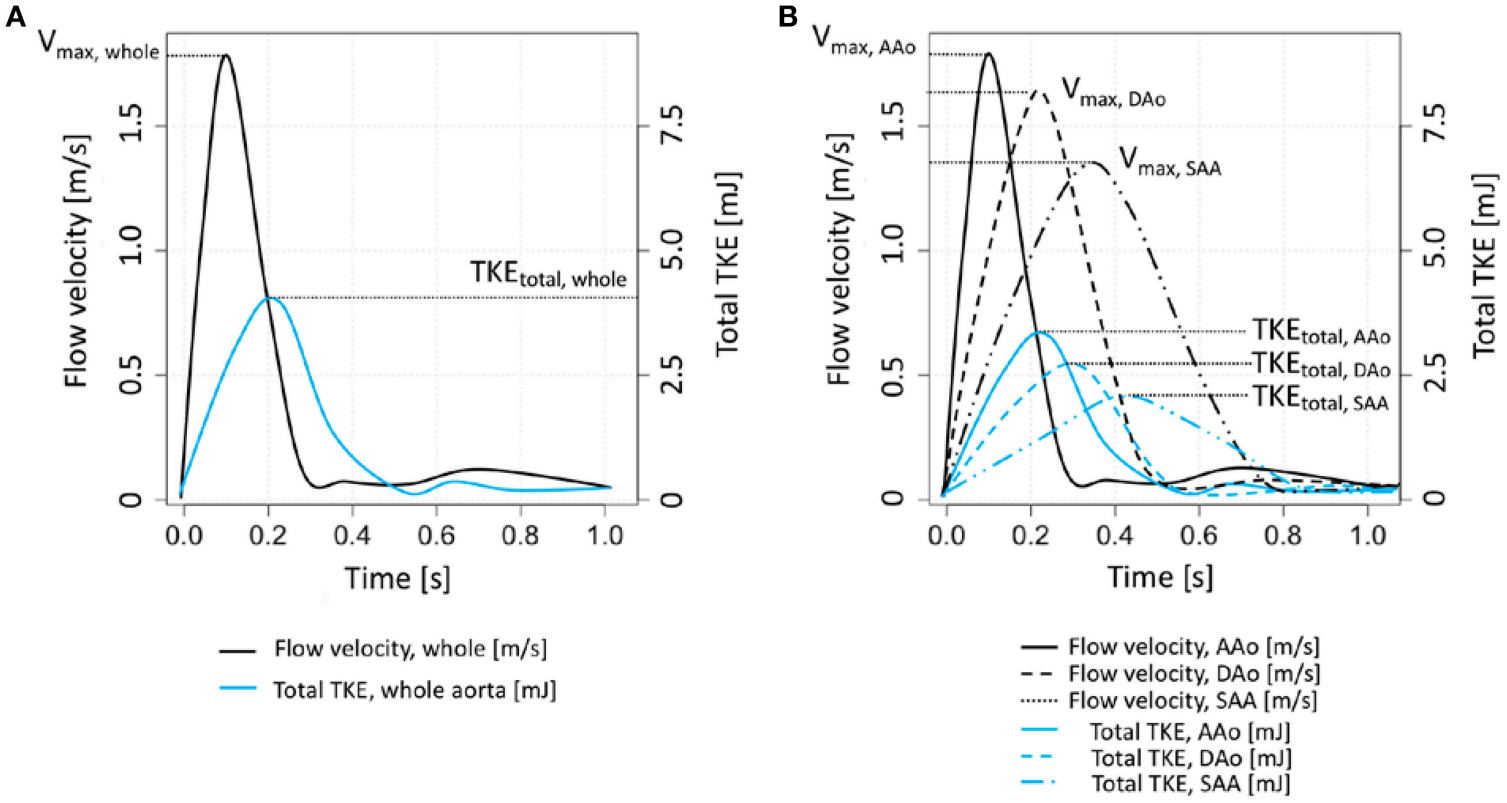
Schematic description of global and regional analysis of flow velocity and TKE. **(A)** Global velocity and TKE analysis. A time point for V_max_, _whole_ was chosen to analyze peak V_avg_, _whole_ and peak V_max_, _whole._ Peak TKE_total_, _whole_ was separately assessed in different time frame with the velocity parameters. **(B)** Regional velocity and TKE analysis. Depending on the region of analysis, separate time frames for V_max_ were selected to assess peak V_avg_ and V_max_ in each aortic segment_._ Separate time frames for each aortic segement were also selected for estimating regional peak TKE_total_. Note that V_max_, _IAA_ and TKE_total_, _IAA_ were omitted from **(B)** for clarity.

Several different measures of turbulence were computed. The TKE per voxel was integrated across the entire aorta as well as across each of the four aortic segments to obtain measures of the total TKE (TKE_total_) globally and regionally in each aortic segment (Figure 2) at two different time points, namely, the time point corresponding to the highest TKE_total_ (“peak TKE_total_”) and the time-point corresponding to the lowest TKE_total_ in diastole (“diastolic TKE_total_”). Additionally, maximum TKE (TKE_max_) and median TKE level (TKE_med_) were also measured in each segment at the time of peak and diastolic TKE_total_ in the segment. Similarly, average velocity (V_avg_) and maximum velocity (V_max_) were also computed in each aorta segment (Figure 2).

### Statistical evaluation

All results are reported as mean ± standard deviation unless otherwise stated. A Shapiro-Wilks test was performed to confirm the normality of the data. A total fifty one inter-group comparisons between the young and old subjects were analyzed with a paired *t*-test using Bonferroni adjusted alpha levels of 0.001 (0.05/51) per test. The correlation between Re and TKE measures were analyzed using Pearson's correlation coefficients. All statistical analyses were performed in Rstudio environment (Rstudio, Boston, MA, USA).

## Results

### Subject characteristics

Of all subjects (*n* = 42), twenty-two were young subjects (23.7 ± 3.0 y/o) and twenty were old subjects (70.9 ± 3.5 y/o). Two (10%) of the old subjects had a history of atrial fibrillation but were in sinus rhythm during MR imaging. Oscillometric blood pressure readings showed that the old subjects had significantly higher diastolic blood pressure (DBP, young = 58.7 ± 3.8 mmHg, old = 71.6 ± 9.3 mmHg,) compared to the young subjects. Seven (35%) and six (30%) of the old subjects were taking hypertension and dyslipidemia medication, repespectively. Further details can be found in Table 1.

**Table 1:** Demographic and basic clinical characteristics of the study subjects.

	**Young**	**Old**	***p*-value**
	**(*n* = 22)**	**(*n* = 20)**	
Age, years	23.7 ± 3.0	70.9 ± 3.5^*^	< 0.001
Height, cm	182.7 ± 6.6	177.5 ± 6.2	0.013
Weight, kg	78.2 ± 10.5	81.0 ± 9.0	0.357
BSA, m^2^	2.0 ± 0.2	2.0 ± 0.1	0.863
SBP, mmHg	110.5 ± 7.1	122.3 ± 16.6	0.007
DBP, mmHg	58.7 ± 3.8	71.6 ± 9.3^*^	< 0.001
Previous cardiac disease			0.427
- No	22 (100.0%)	18 (90.0%)	
- Yes	0 (0.0%)	2 (10.0%)	
Previous lung disease			1.000
- No	20 (90.9%)	19 (95.0%)	
- Yes	2 (9.1%)	1 (5.0%)	
Previous cerebrovascular disease			0.962
- No	22 (100.0%)	19 (95.0%)	
- Yes	0 (0.0%)	1 (5.0%)	
Previous kidney disease			0.962
- No	22 (100.0%)	19 (95.0%)	
- Yes	0 (0.0%)	1 (5.0%)	
Hypertension medication			0.009
- No	22 (100.0%)	13 (65.0%)	
- Yes	0 (0.0%)	7 (35.0%)	
Dyslipidemia medication			< 0.020
- No	22 (100.0%)	14 (70.0%)	
- Yes	0 (0.0%)	6 (30.0%)	
Current smoking			1.000
- No	22 (100%)	20 (100%)	
- Yes	0 (0.0%)	0 (0.0%)	

* *Indicates a statistically significant difference (p < 0.05) between the young and old groups. BSA, body surface area; SBP, systolic blood pressure; DBP, diastolic blood pressure*.

### Anatomical and hemodynamic parameters

Aortic diameter, flow rate, and Reynolds number are shown in Table 2 for both groups. There was no difference in heart rate, SV, or CO between the groups. The maximum flow rates were not significantly different between the groups. The old subjects had larger aortic diameter and lower Reynolds number than the young subjects in all aortic segments, except Reynolds number in IAA.

**Table 2:** Aortic diameter and flow rate.

	**Young**	**Old**	***p*-value**
	**(*n* = 22)**	**(*n* = 20)**	
Heart rate, bpm	63.4 ± 10.4	70.5 ± 12.9	0.053
Stroke volume, mL	68.9 ± 29.8	59.2 ± 23.5	0.254
**CARDIAC OUTPUT, L/min**			
CO	4.3 ± 1.8	4.2 ± 1.9	0.842
**MAXIMUM FLOW RATE, L/m**			
Q_max_, _AAo_	23.5 ± 6.1	20.5 ± 4.4	0.078
Q_max_, _DAo_	17.9 ± 3.1	15.8 ± 3.5	0.045
Q_max_, _SAA_	18.6 ± 3.3	16.4 ± 3.2	0.035
Q_max_, _IAA_	9.9 ± 2.4	9.1 ± 2.7	0.308
**DIAMETER, mm**			
D_AAo_	30.0 ± 3.5	36.4 ± 3.4^*^	< 0.001
D_DAo_	25.3 ± 2.1	31.4 ± 2.4^*^	< 0.001
D_SAA_	22.3 ± 2.2	28.8 ± 2.5^*^	< 0.001
D_IAA_	19.4 ± 2.0	22.2 ± 3.5^*^	< 0.001
**MAXIMUM REYNOLDS NUMBER**			
Re_max_, _AAo_	4408 ± 1084	3177 ± 660^*^	< 0.001
Re_max_, _DAo_	3987 ± 596	2838 ± 568^*^	< 0.001
Re_max_, _SAA_	4707 ± 718	3209 ± 522^*^	< 0.001
Re_max_, _IAA_	2861 ± 596	2289 ± 523	0.002

* *Indicates a statistically significant difference (p < 0.001) between the young and old groups. AAo, ascending aorta; DAo, descending aorta; SAA, suprarenal abdominal aorta; IAA, infrarenal abdominal aorta; CO, cardiac output; Re, Reynolds number*.

**Figure 3:**
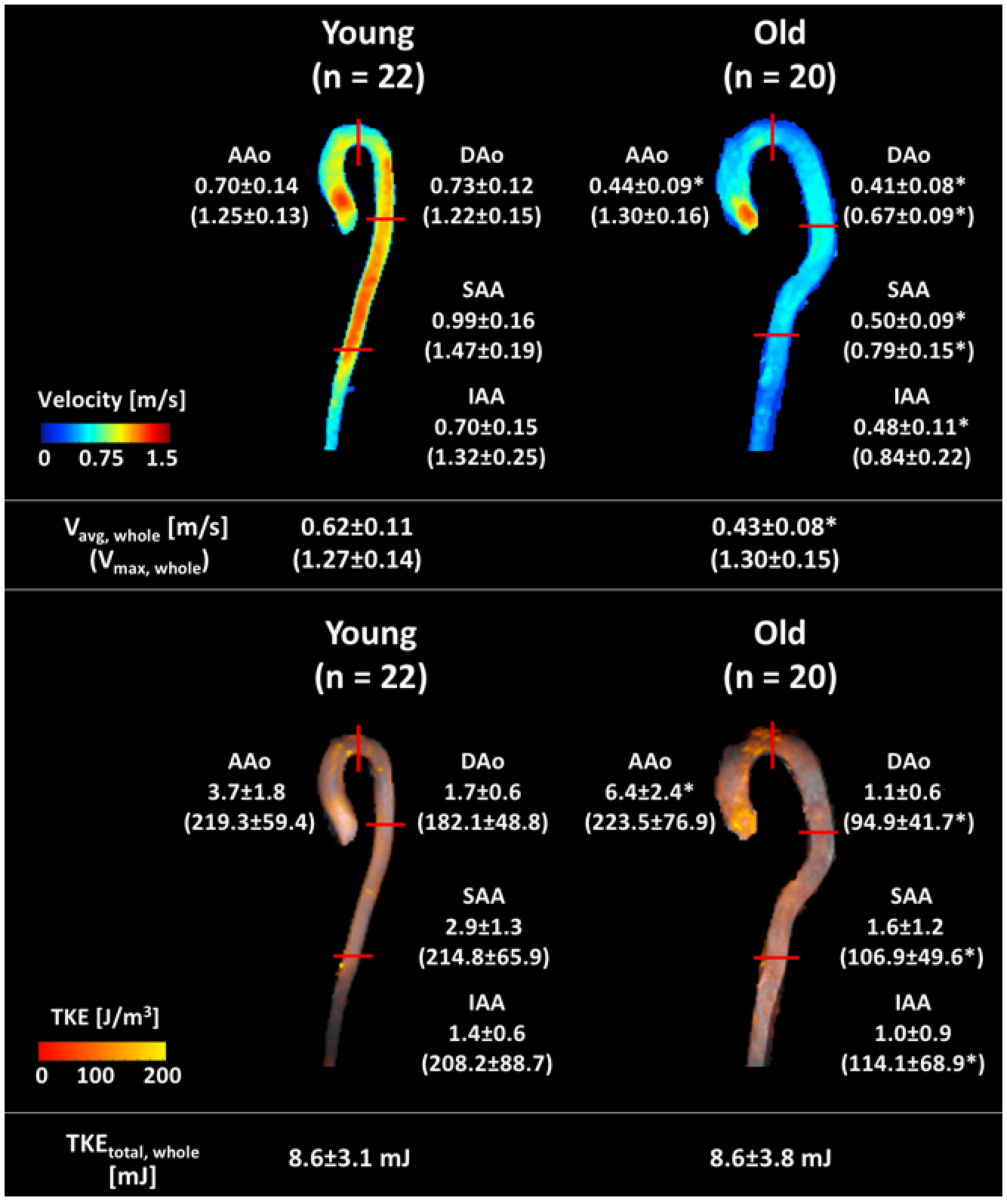
Graphical summary of velocity and TKE parameters in each subject group. Velocity and TKE mapping of a representative subject of each group are shown, with the mean ± *SD* of the group. **Upper** panel shows the regional average velocity (without parentheses, m/s) and maximum velocity (within parentheses, m/s). **Bottom** panel shows the regional peak total TKE (without parentheses, mJ) and the regional peak maximum TKE (within parentheses, J/m^3^). Data are analyzed as described in Figure 2. Red solid lines indicate the region boundaries. ^*^Indicates significant difference with *p* < 0.001 compared to the young group.

Table 3 shows results for estimation of regional velocity and TKE. The old subjects had lower peak average velocities in the whole aorta (V_avg_, _whole_) than the young subjects (young = 0.62 ± 0.11 m/s, old = 0.43 ± 0.08 m/s). Regional analysis showed that the old subjects had significantly lower peak V_avg_ in all regions compared to the young subjects (Figure 3, Table 3). The peak maximum velocity in the ascending aorta (V_max_, _AAo_) was not different between two groups (young = 1.25 ± 0.13 m/s, old = 1.30 ± 0.16 m/s, Figure 3, Table 3). However, the old subjects had lower peak V_max_ for the DAo, SAA, and IAA regions (Figure 3, Table 3).

**Table 3:** Regional flow velocities and TKE parameters.

**Parameter**	**Young**	**Old**	***p*-value**
	**(*N* = 22)**	**(*N* = 20)**	
**PEAK V_avg_, m/s**			
V_avg_, _whole_	0.62 ± 0.11	0.43 ± 0.08^*^	< 0.001
V_avg_, _AAo_	0.70 ± 0.14	0.44 ± 0.09^*^	< 0.001
V_avg_, _DAo_	0.73 ± 0.12	0.41 ± 0.08^*^	< 0.001
V_avg_, _SAA_	0.99 ± 0.16	0.50 ± 0.09^*^	< 0.001
V_avg_, _IAA_	0.70 ± 0.15	0.48 ± 0.11^*^	< 0.001
**PEAK V_max_, m/s**			
V_max_, _whole_	1.27 ± 0.14	1.30 ± 0.15	0.487
V_max_, _AAo_	1.25 ± 0.13	1.30 ± 0.16	0.269
V_max_, _DAo_	1.22 ± 0.15	0.67 ± 0.09^*^	< 0.001
V_max_, _SAA_	1.47 ± 0.19	0.79 ± 0.15^*^	< 0.001
V_max_, _IAA_	1.32 ± 0.25	0.84 ± 0.22^*^	< 0.001
**DIASTOLIC TKE_total_, mJ**			
TKE_total_, _whole_	1.0 ± 0.5	1.3 ± 1.1	0.177
TKE_total_, _AAo_	0.3 ± 0.2	0.7 ± 0.7	0.020
TKE_total_, _DAo_	0.1 ± 0.2	0.2 ± 0.2	0.145
TKE_total_, _SAA_	0.4 ± 0.4	0.3 ± 0.5	0.433
TKE_total_, _IAA_	0.2 ± 0.2	0.2 ± 0.3	0.594
**DIASTOLIC TKE_max_, J/m^3^**			
TKE_max_, _AAo_	61.5 ± 20.1	96.6 ± 94.6	0.119
TKE_max_, _DAo_	74.1 ± 34.3	53.8 ± 29.0	0.046
TKE_max_, _SAA_	88.0 ± 35.2	62.2 ± 23.4	0.008
TKE_max_, _IAA_	79.0 ± 45.7	53.3 ± 24.7	0.028
**DIASTOLIC TKE_med_, J/m^3^**			
TKE_med_, _AAo_	3.2 ± 3.3	4.0 ± 3.3	0.431
TKE_med_, _DAo_	1.7 ± 4.5	2.5 ± 2.7	0.491
TKE_med_, _SAA_	6.3 ± 7.0	2.5 ± 5.0	0.051
TKE_med_, _IAA_	5.3 ± 4.7	2.4 ± 5.6	0.083
**PEAK TKE_total_, mJ**			
TKE_total_, _whole_	8.6 ± 3.1	8.6 ± 3.8	1.000
TKE_total_, _AAo_	3.7 ± 1.8	6.4 ± 2.4^*^	< 0.001
TKE_total_, _DAo_	1.7 ± 0.6	1.1 ± 0.6	0.002
TKE_total_, _SAA_	2.9 ± 1.3	1.6 ± 1.2	0.002
TKE_total_, _IAA_	1.4 ± 0.6	1.0 ± 0.9	0.075
**PEAK TKE_max_, J/m^3^**			
TKE_max_, _AAo_	219.3 ± 59.4	223.5 ± 76.9	0.842
TKE_max_, _DAo_	182.1 ± 48.8	94.9 ± 41.7^*^	< 0.001
TKE_max_, _SAA_	214.8 ± 65.9	106.9 ± 49.6^*^	< 0.001
TKE_max_, _IAA_	208.2 ± 88.7	114.1 ± 68.9^*^	< 0.001
**PEAK TKE_med_, J/m^3^**			
TKE_med_, _AAo_	40.0 ± 11.2	34.3 ± 15.6	0.181
TKE_med_, _DAo_	39.1 ± 10.4	11.9 ± 6.4^*^	< 0.001
TKE_med_, _SAA_	47.6 ± 19.5	16.5 ± 11.2^*^	< 0.001
TKE_med_, _IAA_	39.9 ± 21.3	16.2 ± 9.6^*^	< 0.001

* *Indicates a statistically significant difference (p < 0.001) between the young and old groups. AAo, ascending aorta; DAo, descending aorta; SAA, suprarenal abdominal aorta; IAA, infrarenal abdominal aorta; TKE, turbulent kinetic energy*.

The global and regional diastolic TKE_total_ were not significantly different between the young and old subjects. The diastolic TKE_max_ and TKE_med_ values were not significantly different between the two groups.

Peak TKE_total_, peak TKE_max_, and peak TKE_med_ of all subjects were found to be significantly elevated compared to those at diastole. The peak TKE_total_, _whole_ ranged between 3.8 and 18.8 mJ for the young subjects and 2.7–16.5 mJ for the old subjects. The differences of peak TKE_total_, _whole_ between the groups were not significant (young = 8.6 ± 3.1 mJ, old = 8.6 ± 3.8 mJ, Table 3, Figure 3). However, the old normal subjects had higher peak TKE_total_, _AAo_ than that of the young subjects (young = 3.7 ± 1.8 mJ, old = 6.4 ± 2.4 mJ, Table 3, Figure 3). Regional peak TKE analysis showed no differences in either the maximum or the median level of TKE in AAo (Table 3). In contrast, the old subjects had significantly lower peak TKE_max_ and peak TKE_med_ in the DAo, SAA, and IAA, compared to the young subjects.

### Correlation between reynolds number and TKE

**Figure 4:**
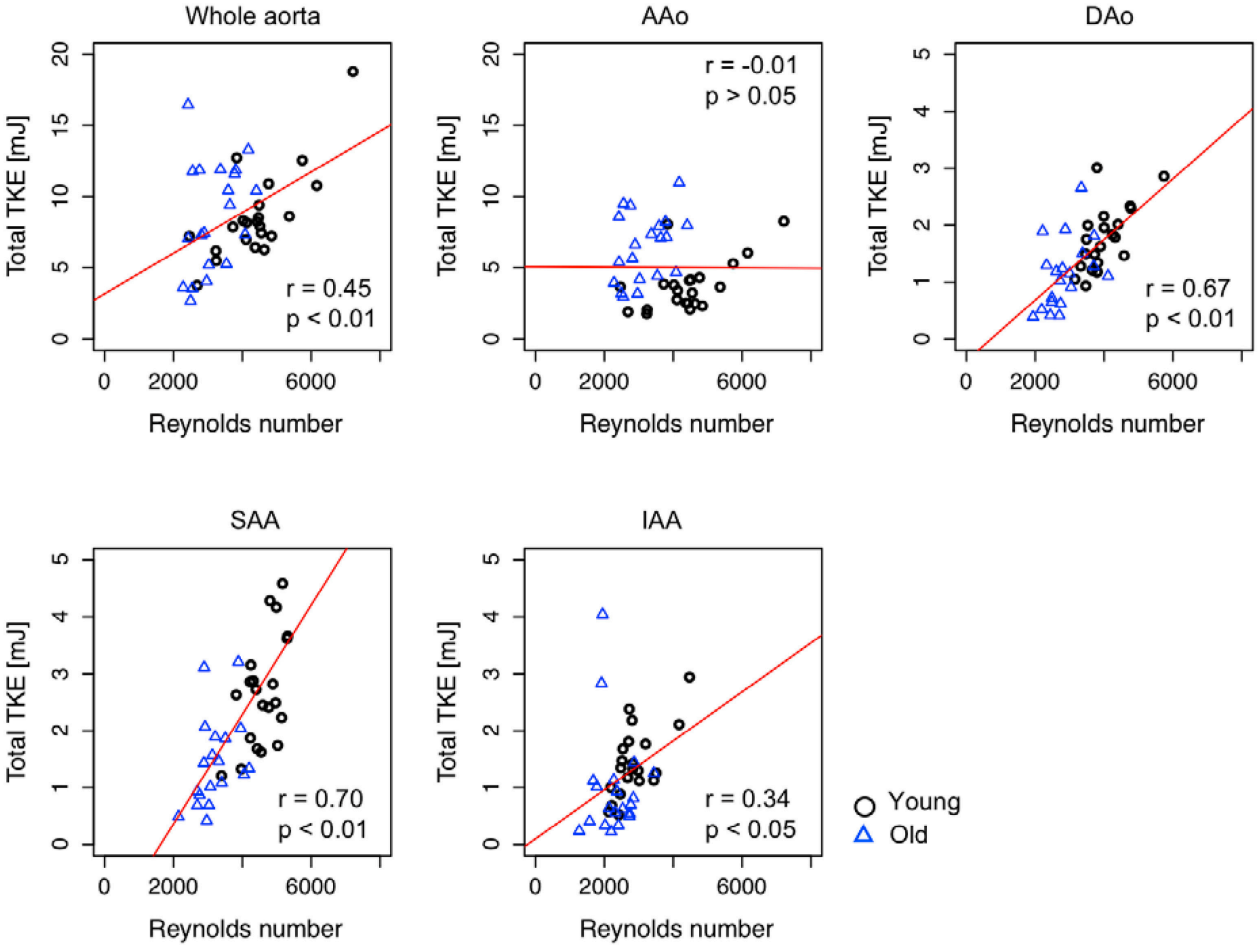
Scatter plot of the relationship between regional peak total TKE and maximum Reynolds number. Pearson correlation coefficient, *r*, between the regional peak total TKE and the maximum Reynolds number were calculated for each region. Red solid line indicates the regression line. *r* and *p* in each plot indicate the Pearson product-moment correlation coefficient and statistical significance. Note that the peak total TKE in the whole aorta (TKE_total_, _whole_) was plotted against the Reynolds number in the ascending aorta (Re_max_, _AAo_) as the Reynolds number is highest in the ascending aorta (as shown in Table 2).

Pearson's correlation analysis showed that peak TKE_total_, _whole_, TKE_total_, _DAo_, TKE_total_, _SAA_, and TKE_total_, _IAA_ were significantly correlated with the maximum Reynolds number for the combined cohort of young and old subjects (Whole aorta, *r* = 0.45; DAo, *r* = 0.67; SAA, *r* = 0.70; IAA, *r* = 0.34, Figure 4, Table 4), while TKE_total_, _AAo_ and TKE_total_, _IAA_ were not correlated with the maximum Reynolds number. Sub-group analyses showed that TKE values for the young subjects were moderately or strongly correlated with the maximum Reynolds number in each analysis region (Whole aorta, *r* = 0.75; AAo, *r* = 0.57; DAo, *r* = 0.68; SAA, *r* = 0.77; IAA, *r* = 0.66). In contrast, in the old subjects, the regional peak total TKE and the maximum Reynolds number were only weakly correlated for DAo (*r* = 0.45) and not significantly correlated for other segments (Table 4).

**Table 4:** Summary of correlations between peak total TKE and maximum Reynolds number.

	**All**	**Young**	**Old**
	**(*n* = 42)**	**(*n* = 22)**	**(*n* = 20)**
TKE_total_, _whole_ vs. Re_max_, _AAo_	0.45 (0.17–0.67)^*^	0.75 (0.16–0.59)^*^	0.33 (−0.13–0.68)
TKE_total_, _AAo_ vs. Re_max_, _AAo_	−0.01 (−0.31–0.30)	0.57 (0.19–0.80)^*^	0.36 (−0.10–0.69)
TKE_total_, _DAo_ vs. Re_max_, _DAo_	0.67 (0.46–0.81)^*^	0.68 (0.52–0.80)^*^	0.45 (0.00–0.74)^*^
TKE_total_, _SAA_ vs. Re_max_, _SAA_	0.70 (0.51–0.83)^*^	0.77 (0.51–0.90)^*^	0.37 (−0.09–0.70)
TKE_total_, _IAA_ vs. Re_max_, _IAA_	0.34 (0.39–0.58)^*^	0.66 (0.32–0.84)^*^	−0.07 (−0.49–0.39)

*Data are Pearson correlation coefficient (r) values. Numbers in parentheses are 95% confidence intervals*.

* *Indicates p < 0.05. AAo, ascending aorta; DAo, descending aorta; SAA, suprarenal abdominal aorta; IAA, infrarenal abdominal aorta; TKE, turbulent kinetic energy; Re, Reynolds number*.

## Discussion

Although the existence of turbulence in aortic blood flow was observed several decades ago (Stein and Sabbah, 1976; Yamaguchi et al., 1983; Hanai et al., 1991), comprehensive descriptions of aortic turbulence have been lacking. The present study measured the extent and degree of turbulence in the aortas of normal subjects by utilizing novel MR flow imaging. All subjects enrolled in this study developed elevated degrees of turbulence throughout the aorta during systole when compared to diastole. TKE_total_ ranged from 2.7 to 18.8 mJ, respectively. Interestingly, while the overall degree of turbulent flow in the whole aorta, as measured by peak total TKE, was not significantly different between young subjects and old subjects, the old subjects had about 73% higher peak total TKE in the ascending aorta compared to the young subjects. This difference was driven by locally increased turbulence in the ascending aorta in the old subjects with larger ascending aorta. Additionally, we found that the Reynolds number, which is commonly used as an indirect indicator of the degree of turbulence, was not strongly correlated to peak total TKE for the old subjects.

The level of TKE_total_ measured here in normal subjects without aortic disease is, as expected, lower than previously reported TKE_total_ in patients with mild to severe aortic stenosis, where the latter group develops TKE_total_ between 13 and 52 mJ (Dyverfeldt et al., 2013; Ha et al., 2016). Compared to previous observations using catheter-based velocity measurements, our study showed that the turbulence level in the normal aorta is an order of magnitude higher (Stein and Sabbah, 1976). We speculate that this could be a result of the underestimation of turbulence with catheter-based velocity measurements. High intensity turbulent flow usually develops in the local boundary of aortic jet flow while lower intensity turbulent flow develops at the center of the flow (Dyverfeldt et al., 2008, 2013; Binter et al., 2015). In addition, turbulent velocity fluctuations are anisotropic, and therefore catheter-based unidirectional velocity measurements based on a single velocity sensing probe in the center of the vessel are unlikely to adequately measure the strongest turbulent fluctuations. In contrast, the 4D Flow MRI technique used in this study permits complete multi-directional and three-dimensional anaysis of turbulent blood flow and has been validated against computational fluid dynamics and particle image velocimetry (Petersson et al., 2010; Arzani et al., 2012; Ha et al., 2016).

The normal aging process brings many changes to the aorta, including increased vessel diameters (Celermajer et al., 1994; O'rourke and Nichols, 2005; Mao et al., 2008; Åstrand et al., 2011; Savji et al., 2013; Dyverfeldt et al., 2014; Van Ooij et al., 2015). While the peak TKE_total_ in the whole aorta was not different between the young and old normal volunteers studied here, peak TKE_total_ in the ascending aorta was higher in the old subjects. As peak TKE_max_ and peak TKE_med_ in the AAo were not significantly different between the two age groups, we reason that age-related aortic dilation is the major cause for the increased peak TKE_total_ in the AAo in the old subjects. The dilation of the AAo results in higher deceleration of the flow distal to the aortic valve and an increased volume for turbulence dissipation, and as such it can be expected to develop higher peak TKE_total_ (Casas et al., 2016). In our study, age-related dilation of the AAo (21.3% increase in diameter) corresponded to increased peak TKE_total_ by 73.0% on average.

Increased vessel diameters in the old subjects were conversely associated with lower peak TKE_total_ in the DAo, SAA, and IAA. In contrast to the characteristic jet flow in the AAo, the flows in the DAo, SAA, and IAA more closely resemble a simple pipe flow system. In these regions, the increased diameter reduces both the peak and average velocity, and consequently suppresses the development of turbulence.

The old subjects in our study had higher systolic blood pressure than the young subjects. Hanai et al. (1991) found a relationship between higher blood pressure and turbulence intensity by using hot-film anemometry in dogs subjected to blood pressure variations induced by an α1-receptor agonist. In agreement with the findings of Hanai et al, we detected higher peak TKE_total_ in the ascending aorta in the old subjects when compared to the young subjects. However, we also note that other TKE parameters, including peak TKE_total_ in the whole aorta, are not different between the two groups. Consequently, the relationship between blood pressure and turbulent blood flow is not clear. Additionally, a growing body of evidence suggests that turbulence plays a role distinct to that played by blood pressure, for example, through the damage it causes to blood constituents and endothelia cells which are not directly coupled to the effects of blood pressure (Mustard et al., 1962; Smith et al., 1972; Stein et al., 1977, 1982; Sallam and Hwang, 1983; Davies et al., 1986; Yoganathan et al., 1986; Lu et al., 2001; Davies, 2009; Yen et al., 2014; Humphrey et al., 2015; Mehta and Tzima, 2016; Wang et al., 2016). Therefore, direct measurements of turbulence can be expected to provide fluid dynamic information beyond that revealed by blood pressure measurements.

The Reynolds number, which includes the ratio between the flow rate and the vessel diameter, has long been used to explain the development of the turbulent flow (Reynolds, 1883; Stein and Sabbah, 1976). Given the technical difficulties associated with measuring turbulence *in vivo*, the Reynolds number has been proposed as an indirect method for assessing the presence of flow instabilities in the aorta (Stalder et al., 2011). However, in our study, indirect assessment of turbulent blood flow using the Reynolds number was only feasible for young subjects. This finding can be explained by the fact that the Reynolds number is defined for simple geometries not comparable to the increasingly tortuous aorta seen in older subjects. The wide range of aortic geometries, mechanical properties, flow pulsatility, and hemorheological parameters in the older subjects appear to prevent the Reynolds number from adequately representing the turbulence *in vivo*.

This study applied the Bonferroni correction to adjust the significance levels (*p* < 0.001) following the large number of comparisons made here. While this method reduces the risk of incorrect rejection of the null hypothesis, it increases the probability of accepting the null hypothesis when the alternative is true. Therefore, it would be meaningful to describe those parameters which were close to but did not reach the adjusted significance level in this study. Compared to the young subjects, the old subjects were shorter (*p* = 0.013, Table 1). The old subjects had higher systolic blood pressure (SBP, young = 110.5 ± 7.1 mmHg, old = 122.3 ± 16.6 mmHg, *p* = 0.007) compared to the young subjects. The maximum flow rates in DAo and SAA were lower in the old subjects (*p* = 0.045 and 0.035, repectively) which could be the reason for lower peak TKE_total_, _DAo_, and peak TKE_total_, _SAA_ (*p* = 0.002) in the old normal subjects compared to the young subjects. Lastly, the diastolic TKE_total_, _AAo_ was larger for the old subjects (*p* = 0.02).

Cardiovascular diseases are localized preferentially to the regions of disturbed flow (Chiu and Chien, 2011). For example, atherosclerosis frequently develops at the bifurcation where the disturbed flow occurs. Therefore, it would be also valuable to assess voxel-wise local analysis of turbulence in the blood flow. Previously, the effect of turbulent flow on the vessel wall has been demonstrated by introducing the near wall turbulence and turbulent wall shear stress (Ziegler et al., 2017). Although this study divided the aorta into four different sub-regions and analyzed volume-wise distribution of TKE, analyzing near-wall TKE and identifying local regions of elevated near wall turbulence would also provide important information for predicting the risk of cardiovascular disease.

This study has several limitations. The study had a cross-sectional design, and only male subjects were analyzed. Further research including both genders is necessary to better understand turbulent flow in normal aortas. In addition, we were unable to perform catheter-based recordings in this study and therefore we cannot directly compare our results against the only other technique that can provide similar measurements.

In conclusion, turbulence develops in the aortas of normal healthy males and is affected by age-related geometrical changes. With age, the turbulence intensity increases in the ascending aorta and decreases in the rest of the aorta. These findings are probably associated with age-related dilation of the aorta. Non-invasive assessment using 4D Flow MRI has the ability to determine what the normal, physiological levels of turbulence are in the aorta, which is a prerequisite for understanding the role of turbulence in the pathophysiology of cardiovascular disease.

## Ethics statement

The ethical review board in Linköping, Sweden approved the study and all subjects gave written informed consent.

## Author contributions

HH analyzed the data and wrote the manuscript, MZ analyzed the data and revised the manuscript, MW acquired the data and revised the manuscript, NB acquired the data and revised the manuscript, C-JC acquired the data and revised the manuscript, ML acquired the data and revised the manuscript, TL acquired the data and revised the manuscript, TE designed the study, analyzed the data and revised the manuscript, PD designed the study, analyzed the data and revised the manuscript. All authors edited the manuscript and have read and approved the final manuscript.

### Conflict of interest statement

The authors declare that the research was conducted in the absence of any commercial or financial relationships that could be construed as a potential conflict of interest.

## Abbreviations

4D Flow MRI: Four dimensional flow magnetic resonance imaging
AAo: Ascending aorta
BSA: Body surface area
CI: Confidence interval
CMR: Cardiovascular magnetic resonance
CO: Cardiac output
DAo: Descending aorta
DBP: Diastolic blood pressure
FOV: Field of view
HSD: Tukey's honestly significant difference
IAA: Infrarenal abdominal aorta
IVSD: Intravoxel velocity standard deviation
Re: Reynolds number
SAA: Suprarenal abdominal aorta
SBP: Systolic blood pressure
TKE: Turbulent kinetic energy
VENC: Velocity encoding range.
